# Supplementary material for: Macrophage migration inhibitory factor is overproduced through EGR1 in TET2low resting monocytes
Source: Commun Biol. 2022 Feb 3;5:110. doi: 10.1038/s42003-022-03057-w (PMC8814058; doi:10.1038/s42003-022-03057-w)
Supplement: Supplementary file 2 — Supplementary Information [file 42003_2022_3057_MOESM2_ESM.pdf]

Macrophage migration inhibitory factor is overproduced through EGR1 in *TET2*^{low} resting monocytes

Elodie Pronier*, Aygun Imanci*, Dorothée Selimoglu-Buet, Bouchra Badaoui, Raphael Itzykson, Thierry Roger, Chloé Jego, Audrey Naimo, Orianne, Maëla Francillette, Marie Breckler, Wagner-Ballon, Maria E. Figueroa, Marine Aglave, Daniel Gautheret, Françoise Porteu, Olivier A. Bernard, William Vainchenker, François Delhommeau, Eric Solary, Nathalie M. Droin.

Supplementary Figures:

Supplementary Figure 1. MIF increase is detected in *Tet2*-KO mice before any blood cell count change

Supplementary Figure 2. *TET2* mutations correlate with a decreased platelet count and better overall survival

Supplementary Figure 3. Increased expression and secretion of MIF in *TET2*-mutated CMML monocytes

Supplementary Figure 4. EGR2 and SP1 recruitment on *MIF* gene proximal promoter

Supplementary Figure 5. MIF promoter is unmethylated in control and CMML monocytes.

Supplementary Figure 6. TET2 interacts with HDAC1 and HDAC2 in primary monocytes

Supplementary Figure 7. EGR1 recruitment to monocyte DNA is modified upon *TET2* mutation

Supplementary Figure 8. Gene Ontology analysis

Supplementary Figure 9. MIF strengthens pathway activation toward monocytic differentiation.

Supplementary Figure 10. Identification of the 7 clusters in CTRL and MIF cultures.

Supplementary Figure 11. Gating strategy for monocyte and granulocyte identification.

Supplementary Figure 12. Uncropped western blots

Supplementary Tables:

Supplementary Table 1: Characteristic of CMML patients whose sorted monocytes were analyzed by RNA sequencing

Supplementary Table 2: Independent cohort of CMML patients used for RT-qPCR analysis of *MIF* gene expression in sorted peripheral blood monocytes

Supplementary Table 3: Characteristic of CMML patients whose bone marrow aspirates were used to quantify MIF

Supplementary Table 4: Cohort of CMML patients whom sorted CD34 cells were analyzed by agilent arrays for gene expression

Supplementary Table 5: Primers and antibodies used in this study

---

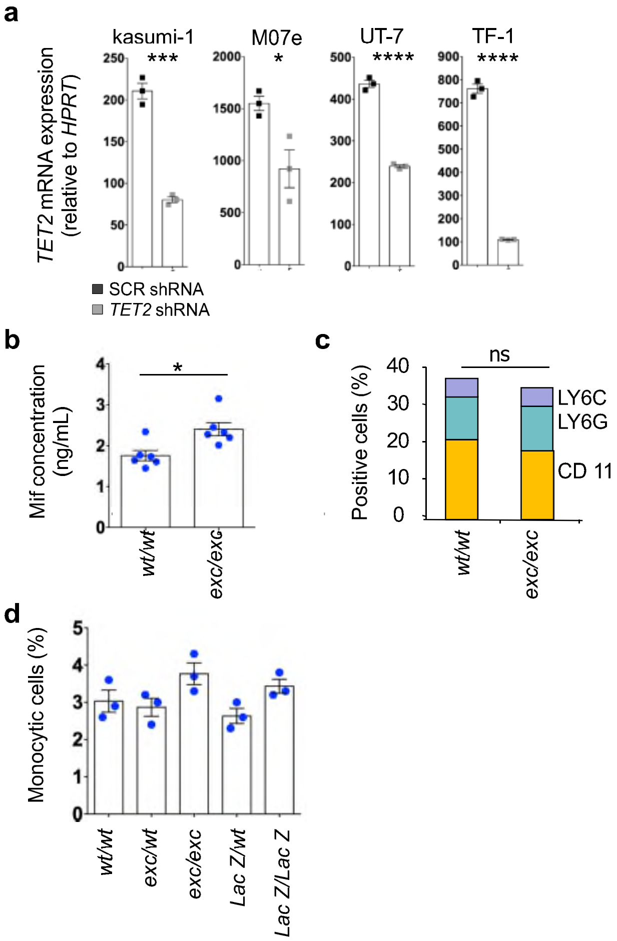

Supplementary Figure 1. MIF increase is detected in *Tet2*-KO mice before any blood cell count change. (a) RT-qPCR analysis of *TET2* mRNA expression in four *TET2*-depleted (*TET2* shRNA, gray bars) and control (SCR shRNA, black bars) human leukemic cell lines. Data are mean +/- SEM of 3 biological replicates. Unpaired t test: * P<0.05; *** P<0.001; **** P<0.0001. **(b)** ELISA quantification of MIF concentration (ng/mL) in bone marrow of young (1-3 months) *Tet2*-knockout K427 animals (wt/wt or $Tet2^{+/+}$ and exc/exc or $Tet2^{-/-}$). Data are mean +/- SEM of 6 different animals. Unpaired t test * P<0.05. (**c**) Flow cytometry analysis of $CD11b^{+}$ (Yellow), $Ly6G^{+}$ (blue) and $Ly6C^{+}$ (gray) cell fractions in the peripheral blood of young (1-3 months), wildtype and $Tet2^{-/-}$ mice. No significant difference between groups of 3 animals in each. **(d)** Monocyte fraction (%) among white blood cells in the peripheral blood of young (1-3 months) in indicated mouse models. No significant difference between groups of 3 animals in each.

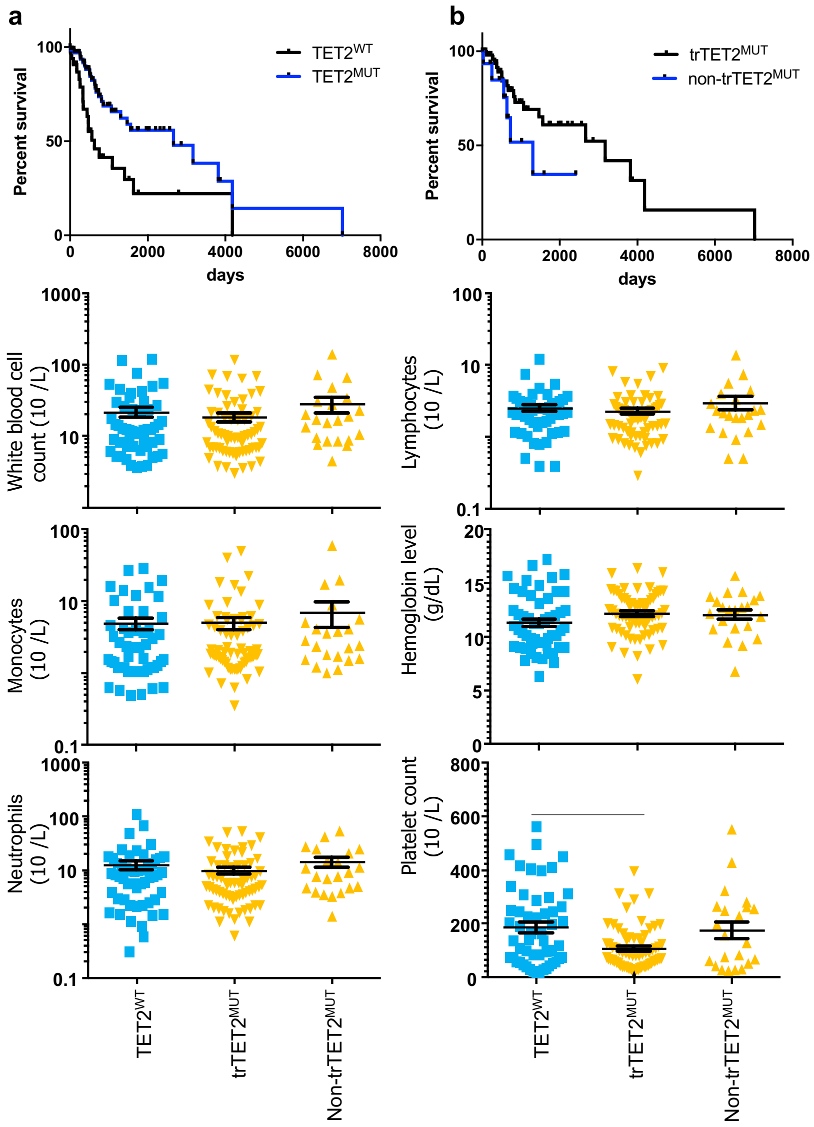

Supplementary Figure 2. *TET2* mutations correlate with a decreased platelet count and better overall survival. **(a)** Kaplan–Meier estimates of overall survival of (*TET2*^{WT}, n=52) and (*TET2*^{MUT}, n=89) CMML patients; Log-rank (Mantel-Cox test); P=0.0029; **(b)** Kaplan–Meier estimates of overall survival of CMML patients with tr*TET2*^{Mut} (n=67) and non-tr*TET2*^{Mut} (n=22); Log-rank (Mantel-Cox test); P=0.1262. **(c)** Peripheral blood parameters in CMML patients without TET2 mutation (*TET2*^{WT}, n=52, for 4 patients we do not have clinical data) or with (tr*TET2*^{Mut} (n=67, for 1 patient we do not have clinical data), non-tr*TET2*^{Mut} (n=22). The platelet count was significantly lower in tr*TET2*^{MUT} patients. Data are mean +/- SEM of indicated biological samples. Dunnett's multiple comparison test using *TET2*^{WT} as control, *** P<0.001.

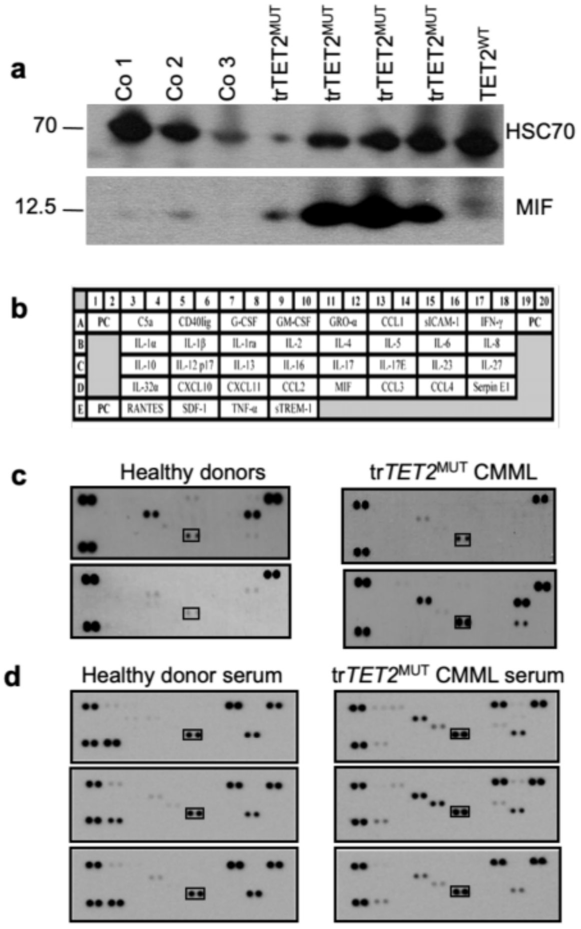

Supplementary Figure 3. Increased expression and secretion of MIF in *TET2*-mutated CMML monocytes. (a) Immunoblot analysis of MIF expression in sorted peripheral blood monocytes collected from three age-matched healthy donors (Co), 4 tr*TET2*^{MUT} and 1 *TET2*^{WT} CMML patients. HSC70 (sc-7298, Santa Cruz), loading control. **(b)** Cytokine array map; **(c)** Sorted monocytes from 2 age-matched healthy donors and 2 tr*TET2*^{MUT} CMML patients were incubated in RPMI medium for 18 hours before cytokine array analysis of culture supernatant. MIF is framed by a rectangle. **(d)** Cytokine array analysis of the serum collected from 3 age-matched healthy donors and 3 tr*TET2*^{MUT} CMML patients. MIF is framed by a rectangle.

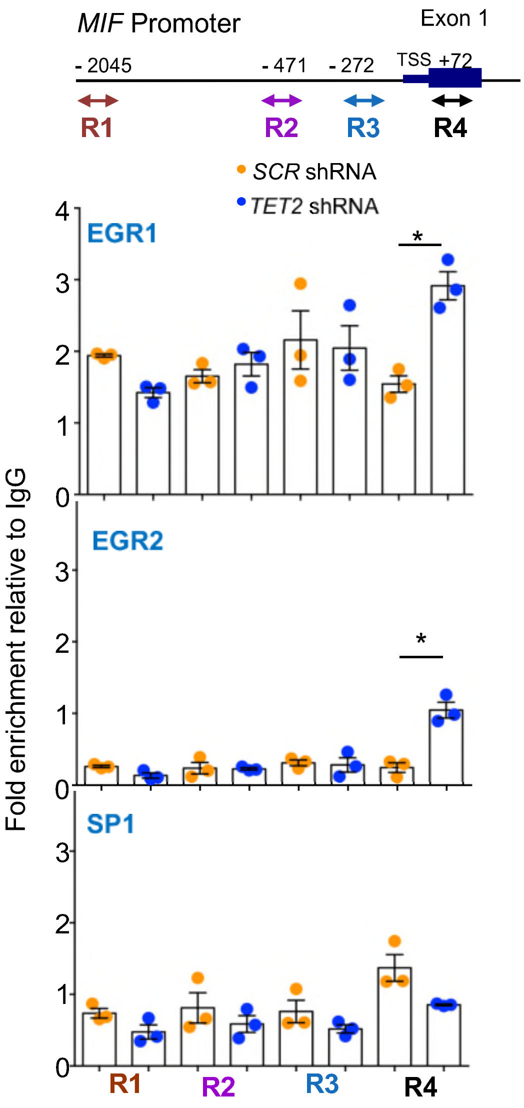

Supplementary Figure 4. EGR2 and SP1 recruitment on *MIF* gene proximal promoter. Schematic representation of *MIF* gene promoter; quantitative ChIP-PCR analyses were performed in kasumi-1 cells infected by *SCR* (yellow) or *TET2* (blue) shRNAs by using a set of primers targeting regions located in distal (R1), intermediate (R2), proximal (R3) promoter and in exon 1, closed to the TSS (R4). EGR1 (sc-110, Santa Cruz), EGR2 (sc-20690, Santa Cruz) and SP1 (#39058, Active Motif) associated DNA was immunoprecipitated. Results are expressed as fold enrichment relative to DNA immunoprecipitated with control immunoglobin G (IgG). Data are mean +/- SEM each sample run in triplicate. Unpaired t test, * P<0.05.

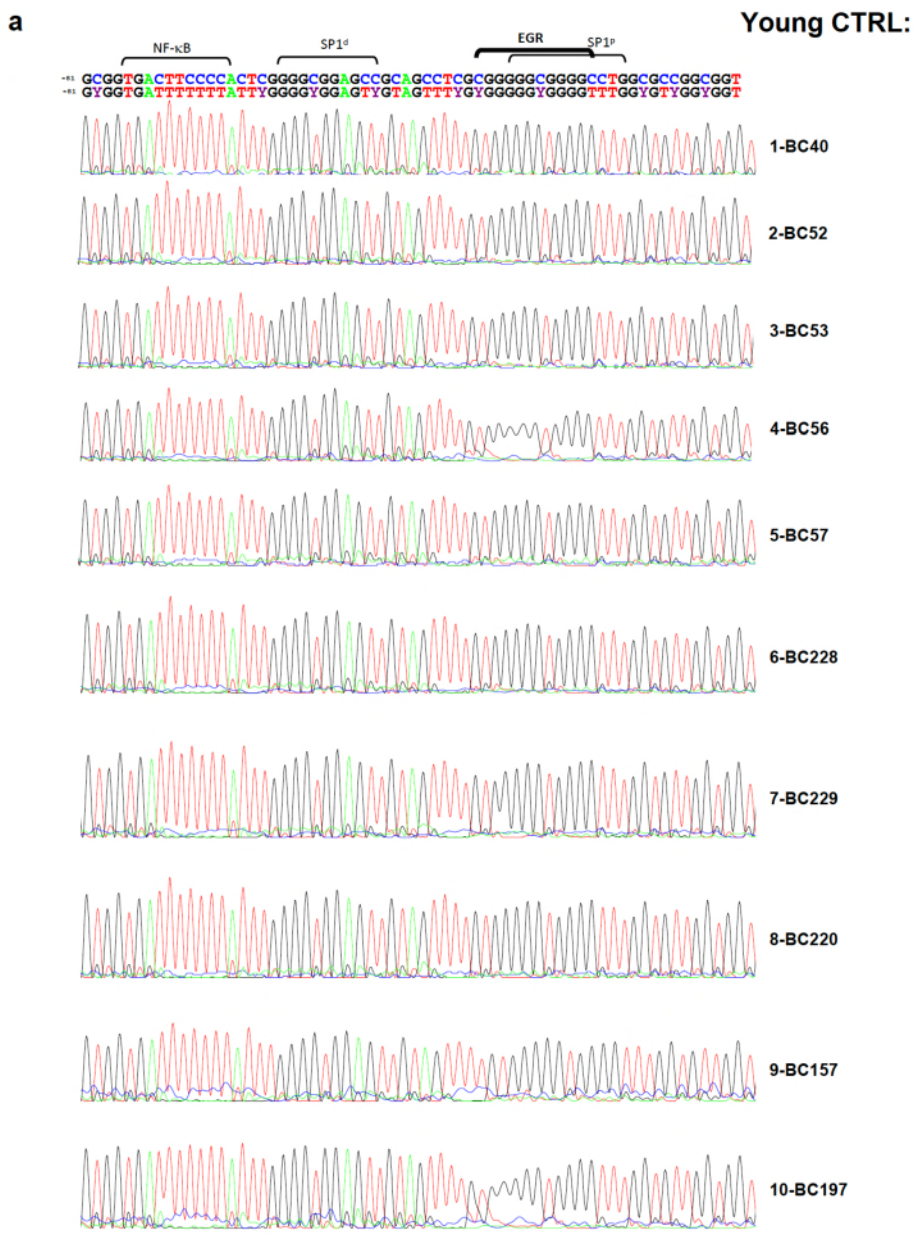

a
Young CTRL:
NF-κB
SP1d
EGR
SP1p
GCGGTGACTTCCCCACTCGGGGCGGAGCCGCAGCCTCGCGGGGGCGGGGCCTGGCGCCGGCGGT
GYGGTGATTTTTTTTATTYGGGGYGGAGTYGTAGTTTYGYGGGGGYGGGGTTTGGYGTYGGYGGT
1-BC40
2-BC52
3-BC53
4-BC56
5-BC57
6-BC228
7-BC229
8-BC220
9-BC157
10-BC197

b

Age-matched CTRL:

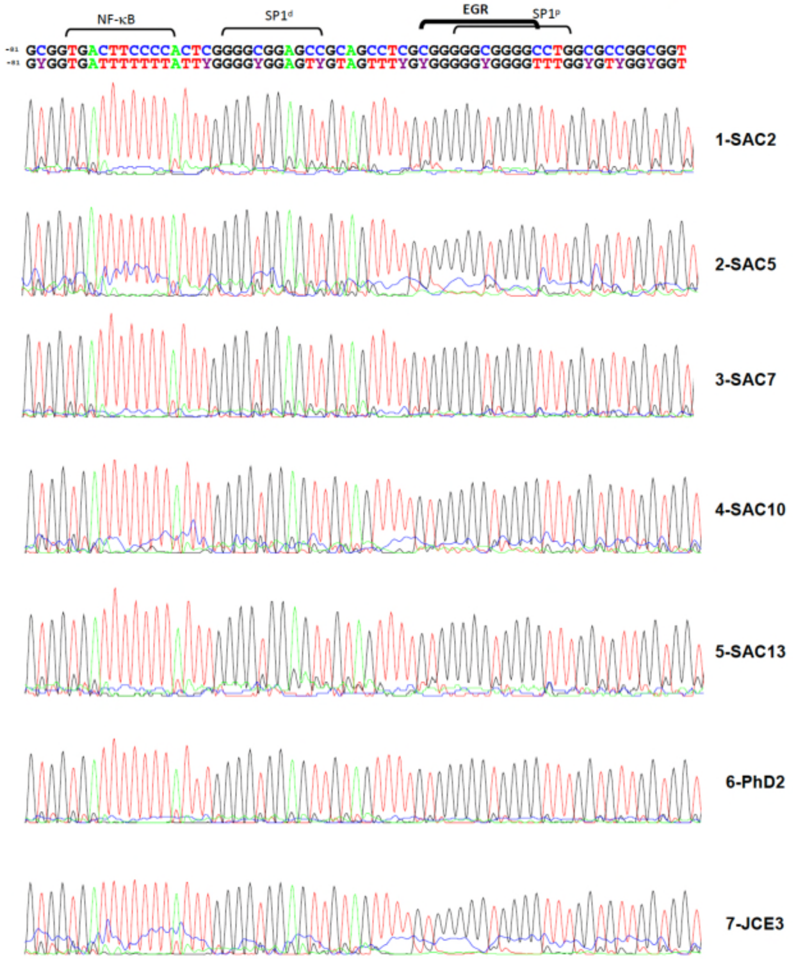

c

TET2^{MUT} CMML:

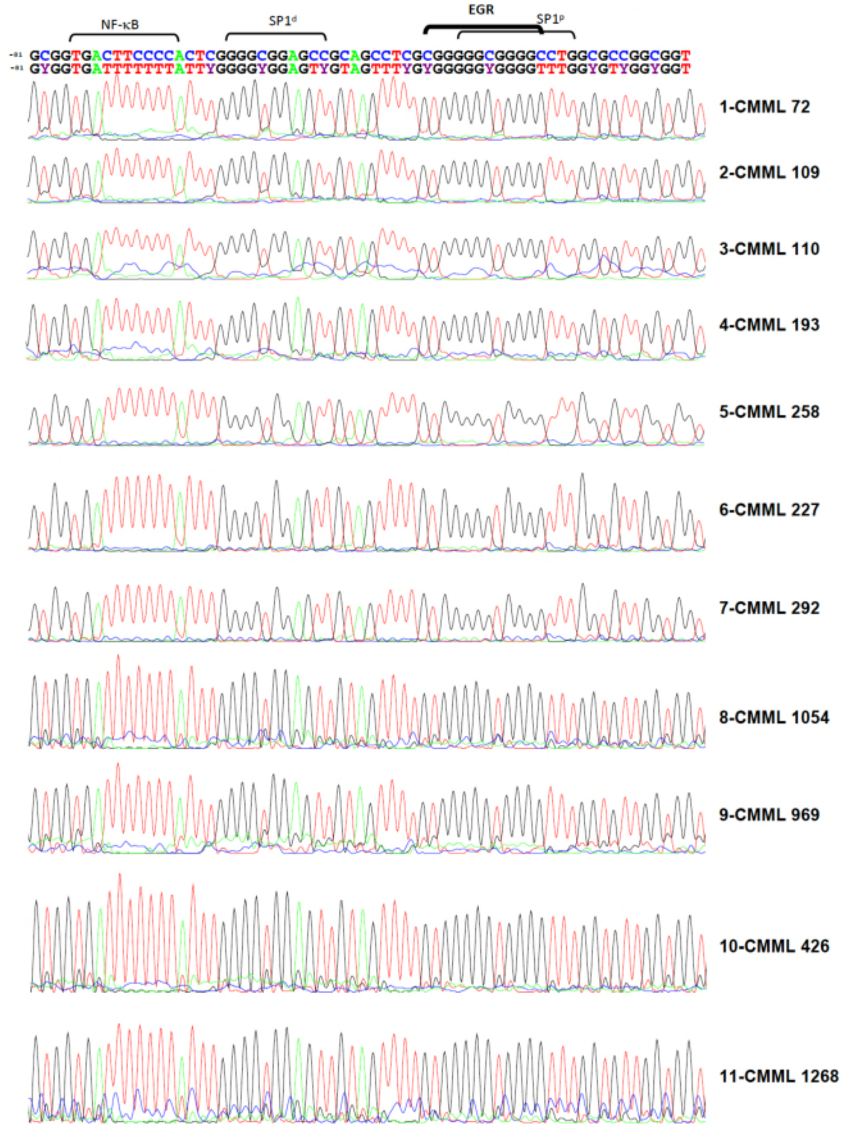

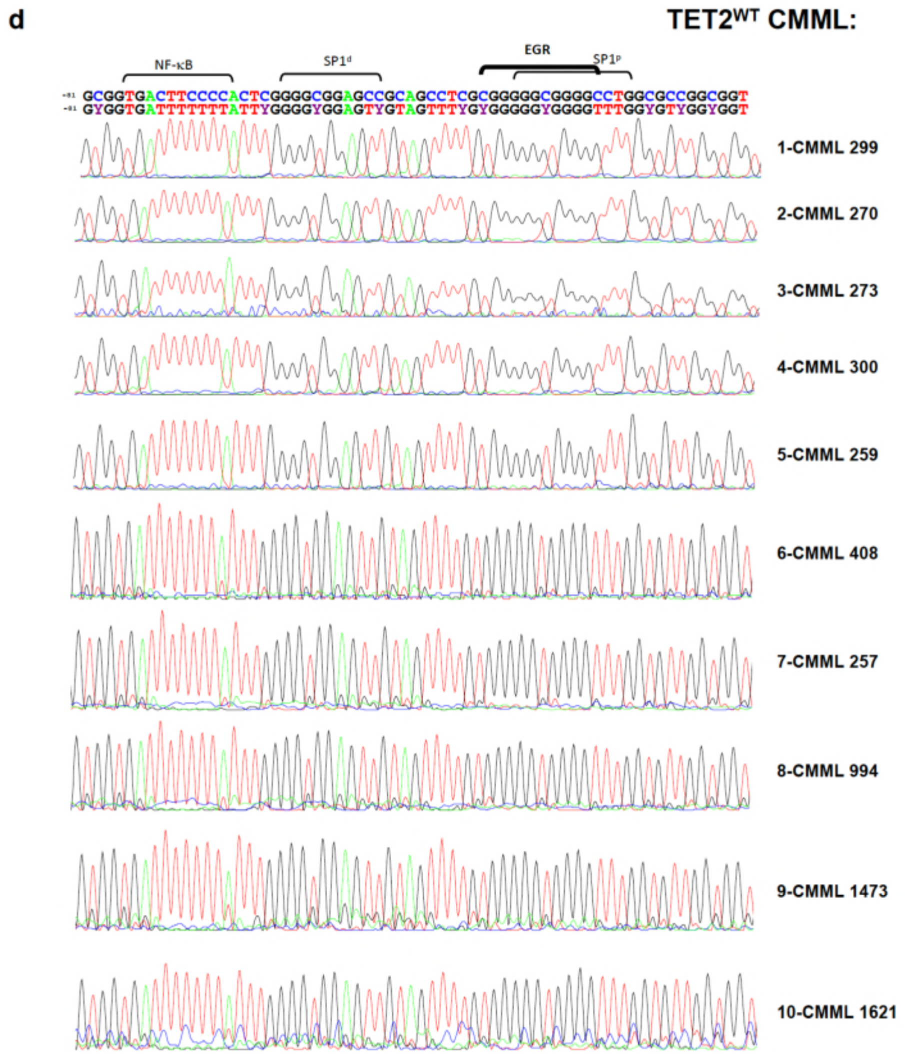

Supplementary Figure 5. MIF promoter is unmethylated in control and CMML monocytes. Sanger sequencing of the bisulfite-modified *MIF* promoter in CD14+ monocytes from 10 young controls **(a)**, 7 age-matched donors **(b**, elderly), 11 wild-type TET2 CMML **(c)** and 10 mutated TET2 CMML **(d)**.

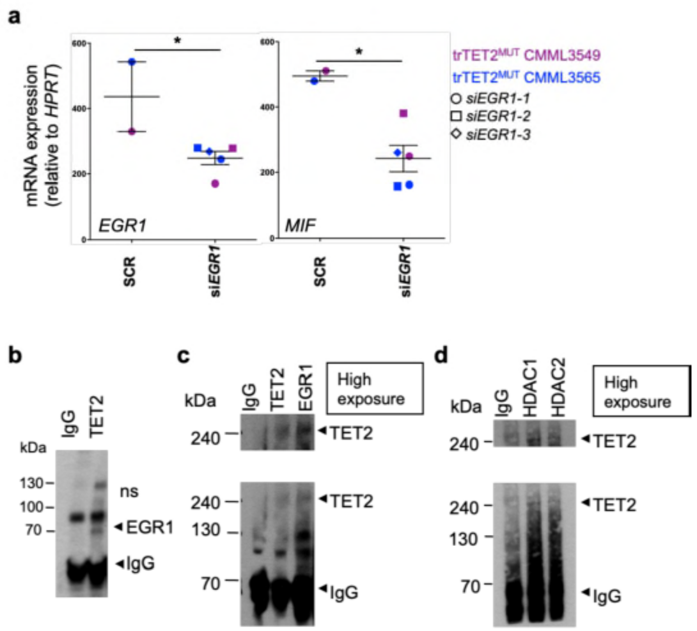

Supplementary Figure 6. TET2 interacts with HDAC1 and HDAC2 in primary monocytes.

(a) siRNA-mediated down-regulation of *EGR1* in trTET2^{MUT} monocytes isolated from CMML3549 (purple) and CMML3565 (blue) with 2 and 3 sets of *EGR1*-siRNA respectively. RT-qPCR analysis of *EGR1* and *MIF* mRNA expression normalized to *HPRT*, tested in triplicate. Data are mean +/- SEM of each sample run in triplicate. Unpaired Students-t test using control monocytes transfected with SCR as control, * P<0.05. **(b, c)** Co-immunoprecipitation experiments in sorted healthy donor monocytes. **(b)** An anti-TET2 (sc-136926) or a control IgG were used for IP, followed by immunoblotting with anti-EGR1 (sc-110). **(c)** An anti-TET2 (sc-136926), an adnti-EGR1 (sc-110) or a control were used for IP, followed by immunoblotting with an anti-TET2 antibody (upper panel is a higher exposure version of the same blot). **(d)** Immunoprecipitation was performed in sorted healthy donor monocytes using an anti-HDAC1 (#39531, Active Motif) or an anti-HDAC2 (#39533, Active Motif) or a control IgG followed by immunoblotting with anti-TET2 antibody (sc-136926).

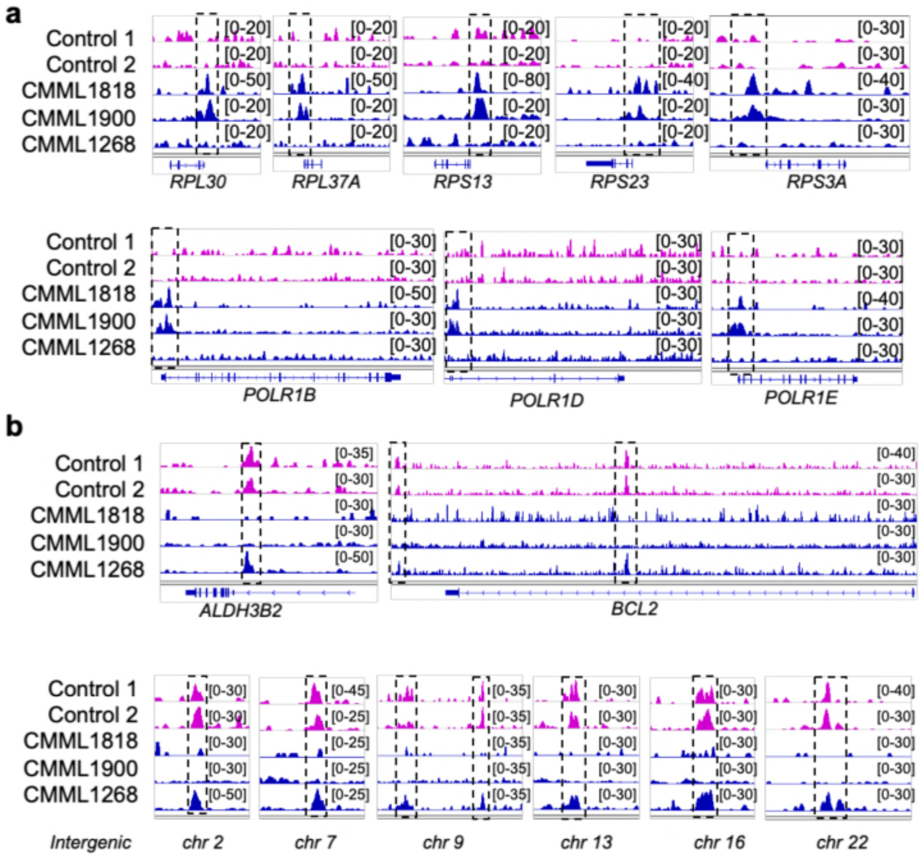

Supplementary Figure 7. EGR1 recruitment to monocyte DNA is modified upon *TET2* mutation. ChIP-seq experiments were performed as described in Figure 6 using an anti-EGR1 antibody in sorted peripheral blood monocytes from 2 healthy donors (controls) and three CMML patients **(a)** Peak calling for EGR1 on indicated genes in controls (pink) and CMML samples (dark blue). **(b)** Peak calling for EGR1 on indicated intergenic regions in controls (pink) and CMML samples (dark blue).

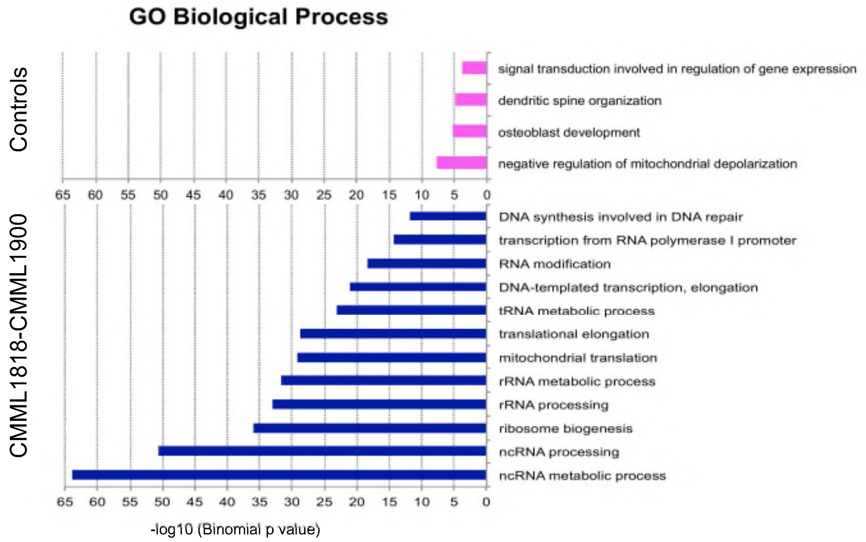

Supplementary Figure 8. GO (Gene Ontology) biological processes detected by analyzing EGR1 ChIP-sequencing data with GREAT (Genomic Regions Enrichment of Annotations Tool: http://great.stanford.edu/public/html).

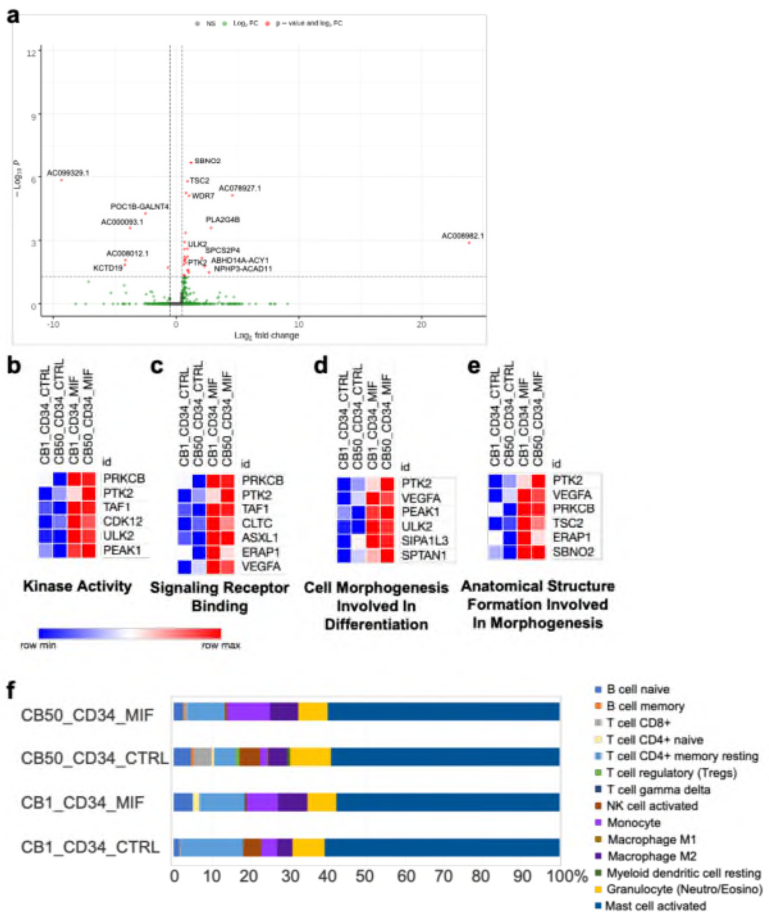

Supplementary Figure 9. MIF strengthens pathway activation toward monocytic differentiation. Cord blood CD34^{+} cells were cultured in medium with SCF, FLT3L, IL-3 and G-CSF with or without MIF during 48h and studied by RNA-seq. **(a)** Volcano plot representation of differentially expressed genes in CTRL versus MIF cultures. The name of differentially deregulated genes is indicated. **(b, c)** GO Molecular Function analysis of differentially deregulated genes represented by heatmap. **(d, e)** GO Biological Process analysis of differentially deregulated genes represented by heatmap. **(f)** CIBERSORT analysis to estimate the abundance of hematopoietic cell types in the mixed cell population, using gene expression data.

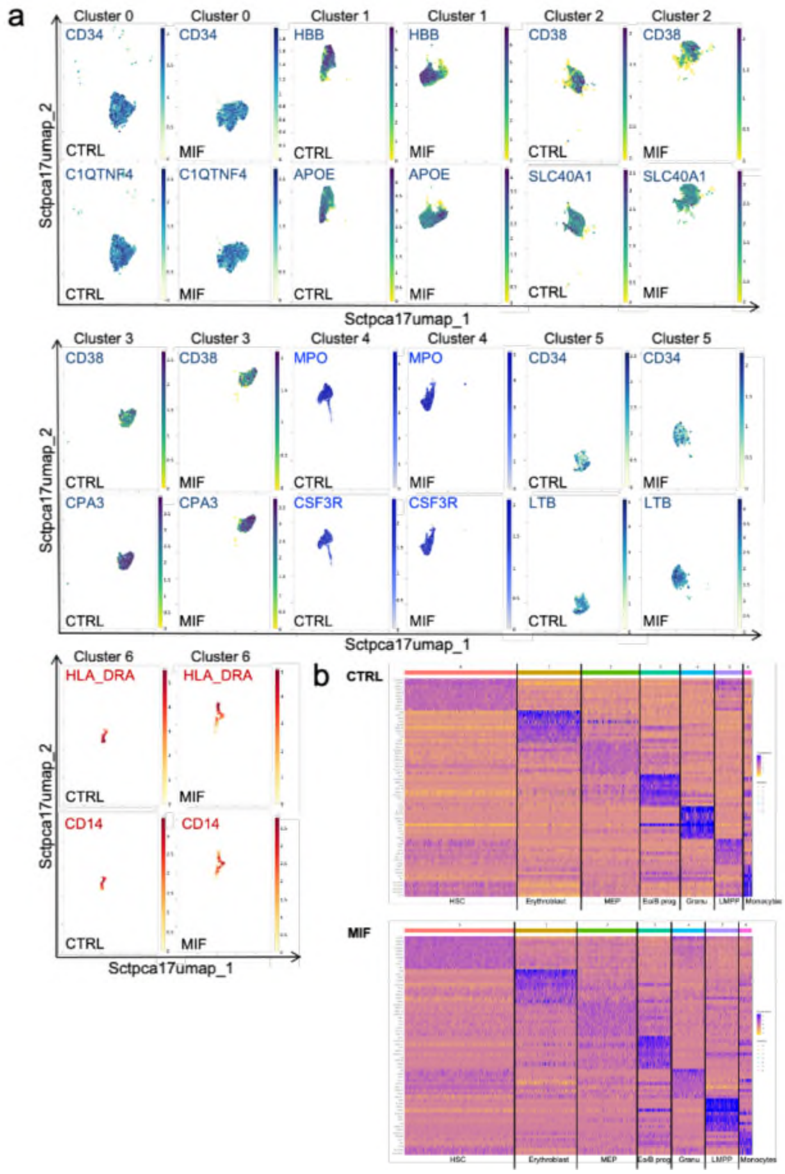

Supplementary Figure 10. Identification of the 7 clusters in CTRL and MIF cultures. Cord blood CD34⁺ cells from CB50 were cultured in medium with SCF, FLT3L, IL-3 and G-CSF with or without MIF during 7 days and studied by scRNA-seq. **(a)** Umaps of 2 discriminant genes in each of the 7 clusters identified in CTRL and MIF cultures. Each cluster was identified thanks to *CD34*, *C1QTNF4*, *HBB*, *APOE*, *CD38*, *SLC40A1*, *CPA3*, *MPO*, *CSF3R*, *LTB*, *HLA-DRA* and *CD14* gene expressions. **(b)** Heatmaps of the 10 top genes in each of the 7 clusters identified in CTRL and MIF cultures. cluster 0: HSC (hematopoietic stem cells); cluster 1: erythroblast; cluster 2: MEP (megakaryocyte–erythroid progenitor cells); cluster 3: Eo/B progenitors (eosinophil/basophil progenitors); cluster 4: granulocytes; cluster 5: LMPP (lymphoid-primed multipotent progenitors) and cluster 6: monocytes.

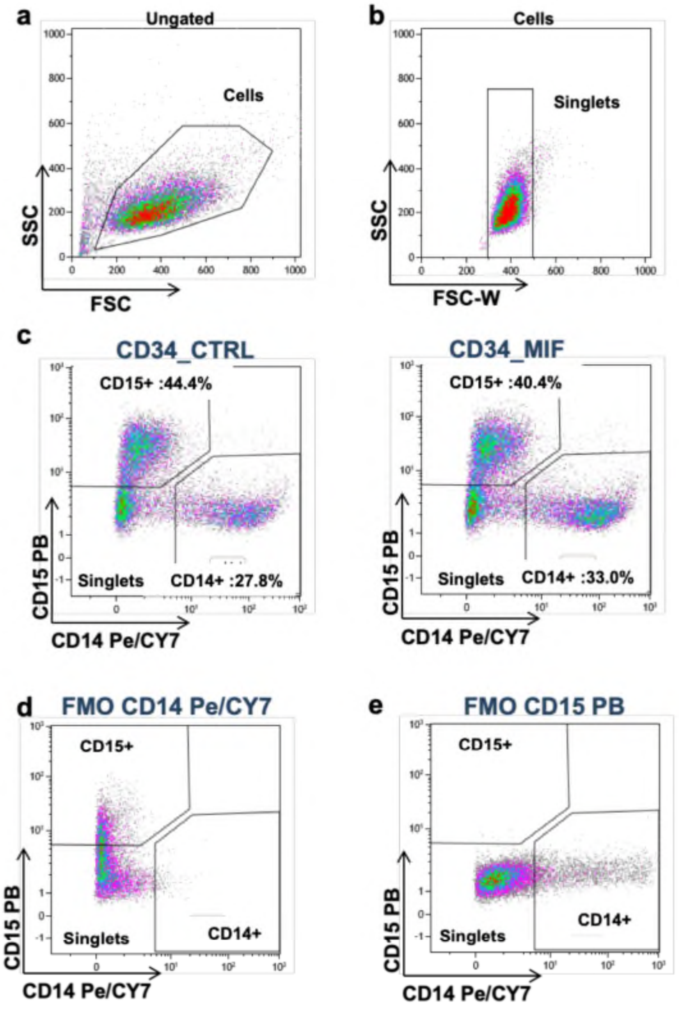

Supplementary Figure 11. Gating strategy for monocyte and granulocyte identification. Differentiation of cord blood CD34$^+$ cells during 7 to 9 days with stem cell factor (SCF), interleukin-3 (IL-3), Fms-related tyrosine kinase 3 ligand (FLT3L), granulocyte-colony stimulating factor (G-CSF) in the presence or the absence of 20 ng/mL MIF before flow cytometry analysis. Using forward (FSC) and side scatter (SSC-A) (**a**, **b**), dead cells and doublets were eliminated. On the remaining cultured cells, differentiated granulocytic population is identified by CD15$^+$ expression, while monocytic population is identified by CD14$^+$ expression on a CD14/CD15 dot plot (**c**). CD15 and CD14 gate positions were determined using Fluorescence minus one (FMO) controls (**d**, **e**).

Uncropped blots related to Figure 1e

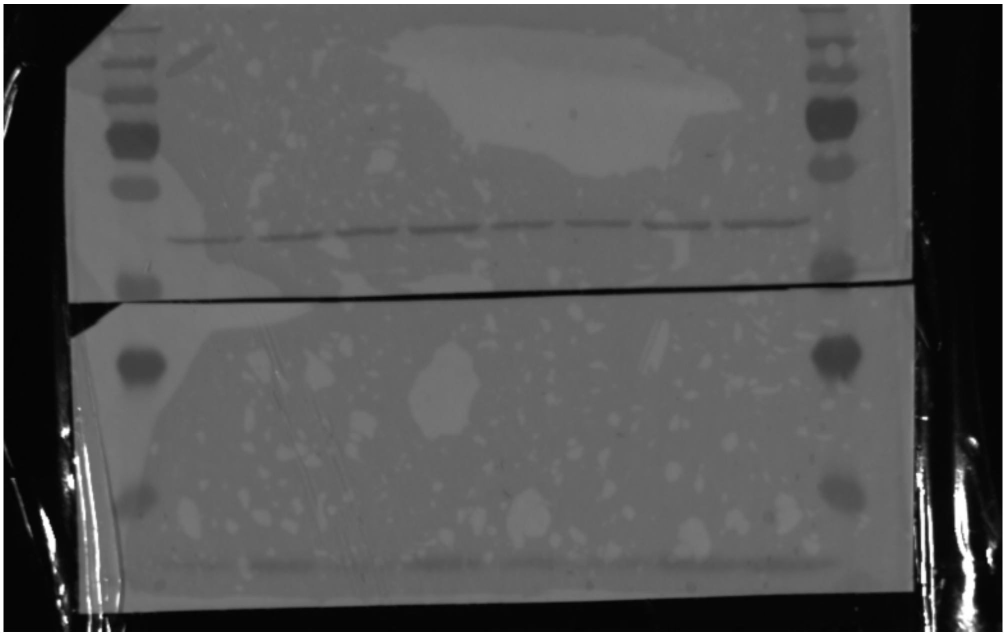

anti-ACTIN

anti-MIF

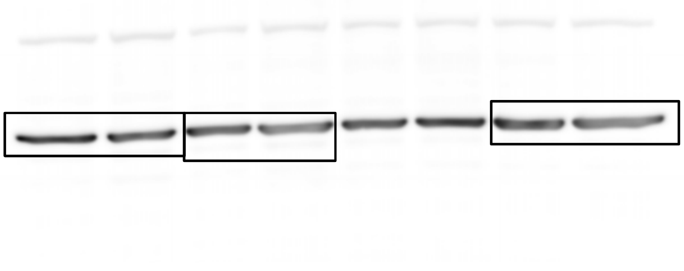

anti-ACTIN

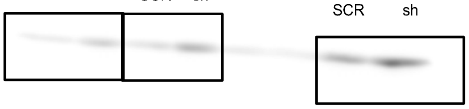

anti-MIF

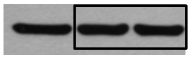

anti-ACTIN

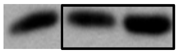

anti-MIF

Uncropped blots related to Figure 5d

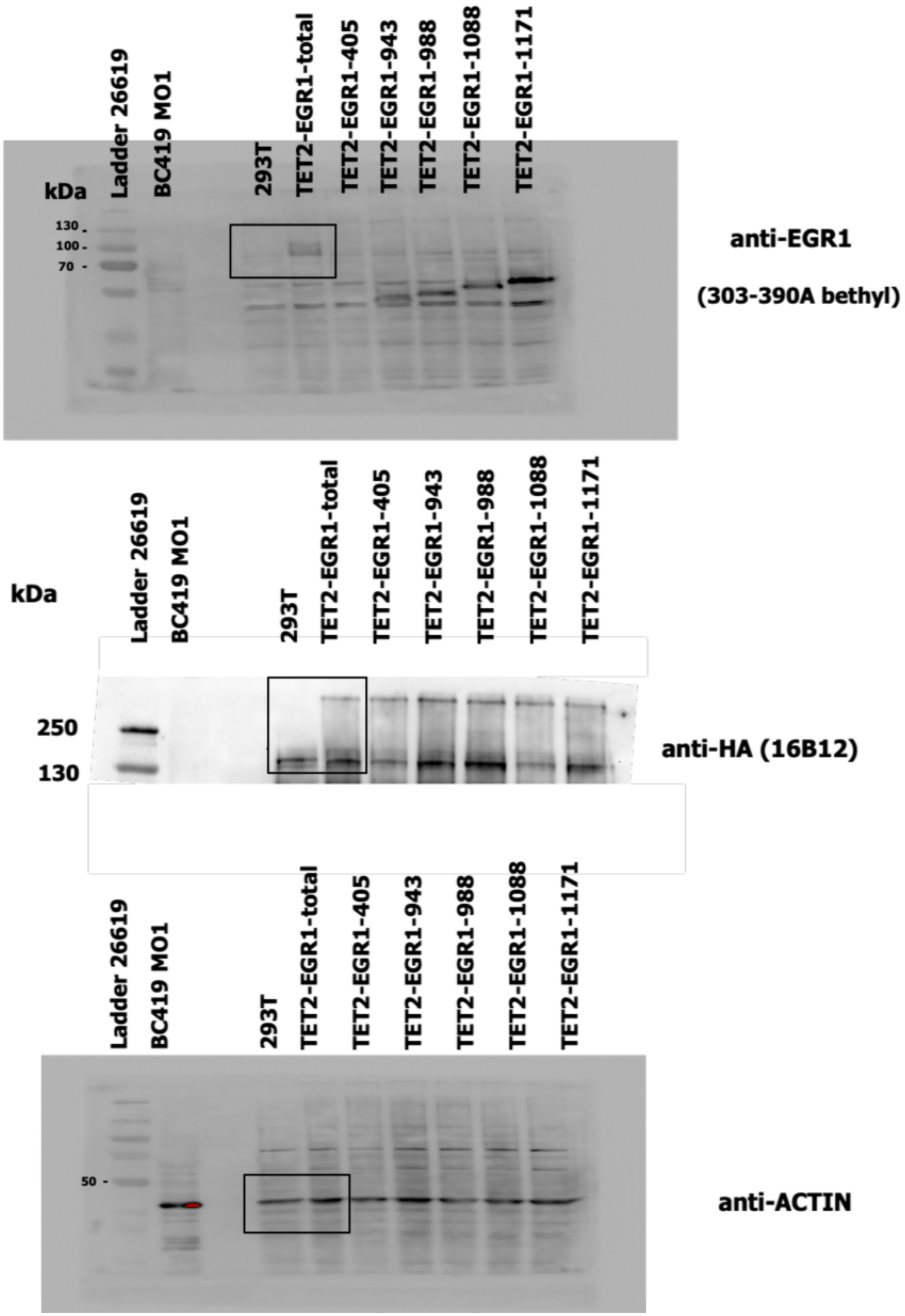

Uncropped blots related to Figure 5e

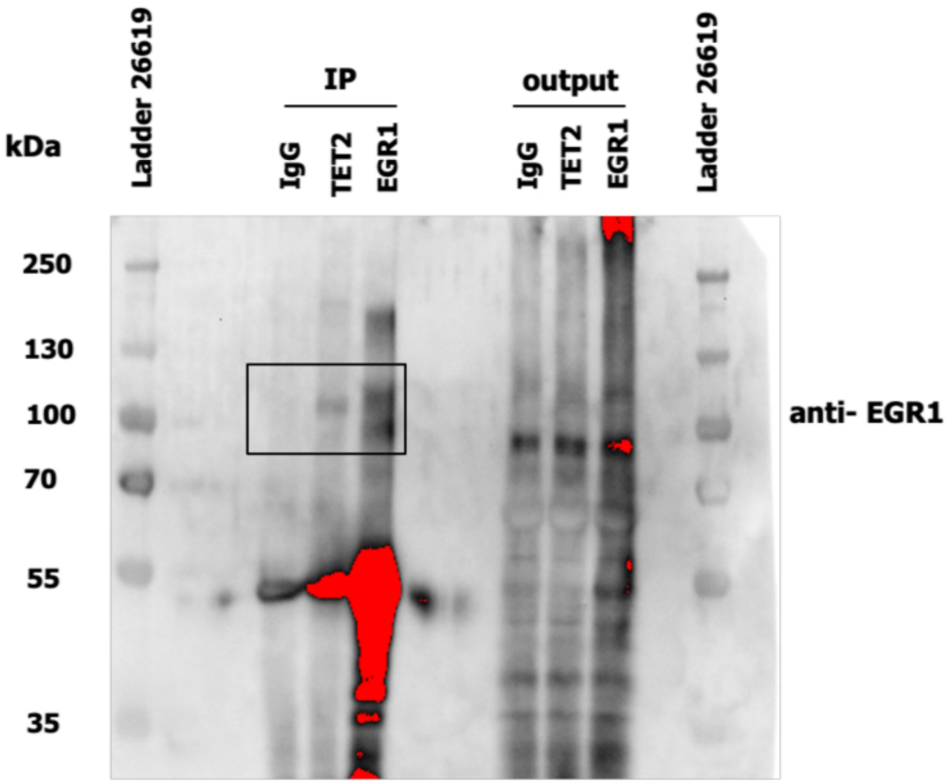

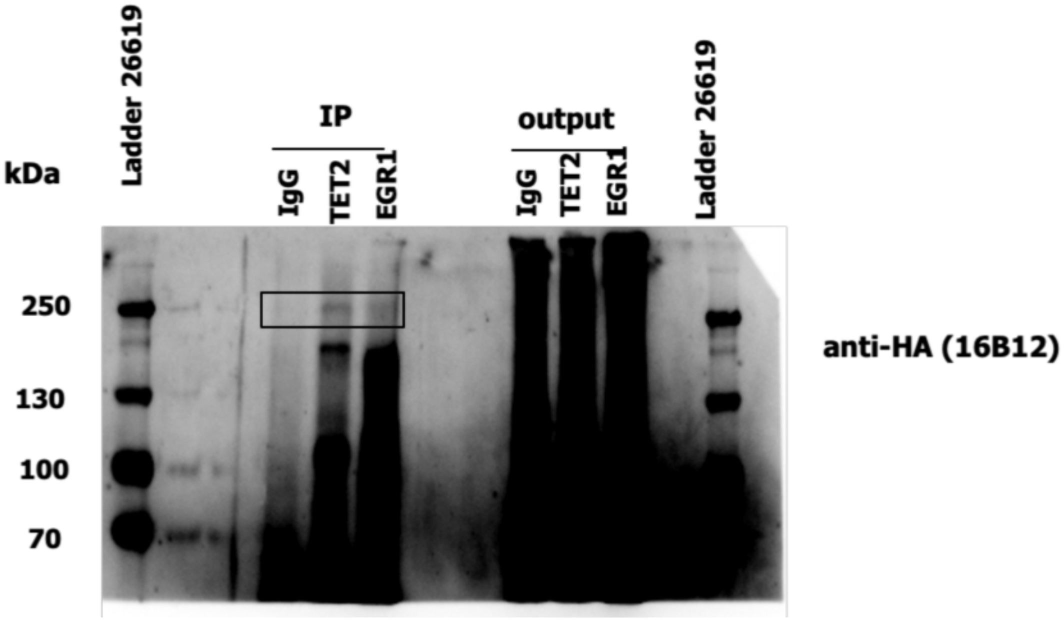

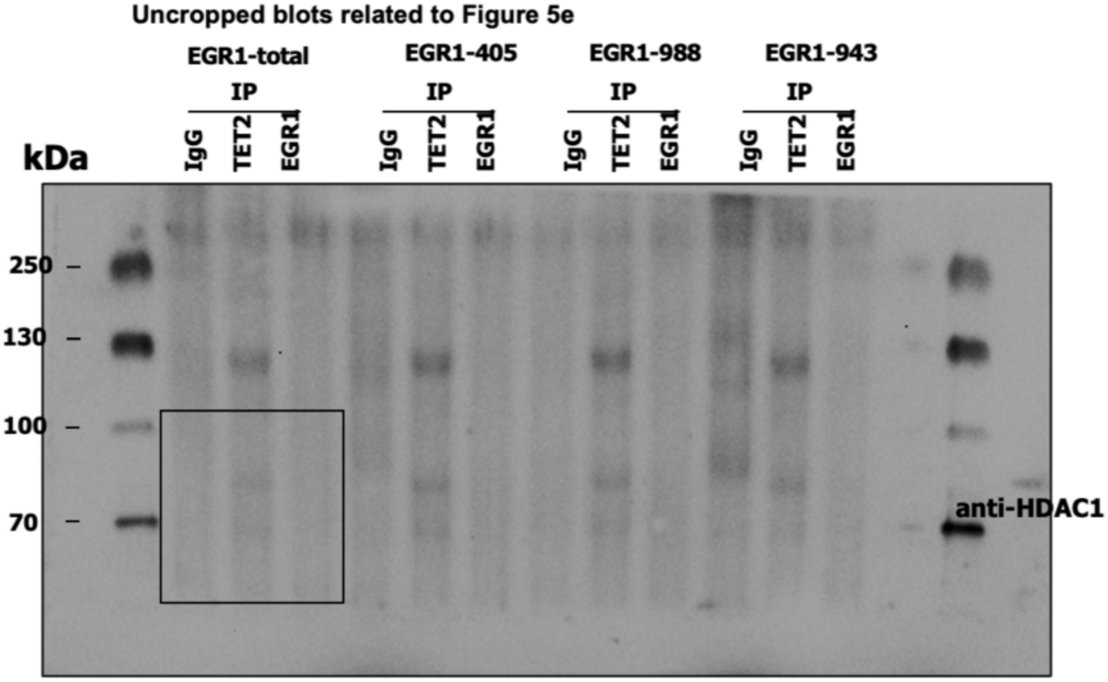

Supplementary Figure 12. Uncropped western blots

Supplementary Table 1: Characteristic of CMML patients whose sorted monocytes were analyzed by RNA sequencing.

**CMML**	**TET2 wild type**	**TET2 mutated**
Number of cases	17	43
Mean age in years (range)	68.1 (53-79)	71.3 (45-87)
Sex ratio M/F	10/7/0	30/10/3
CMML 0/1/2	5/6/5	16/11/9
Proliferative/Dysplastic	11/4	29/10
Leucocytes, mean.10^9/L (range)	35.1 (2.7-119)	27.0 (4.8-110.4)
Monocytes, mean.10^9/L (range)	10.4 (1.7-55.2)	7.9 (1.1-67.0)
Platelet count, mean.10^9/L (range)	137.7 (18-417)	123.4 (25-394)
Hemoglobin level, mean g/dL (range)	9.2 (6.3-13.9)	11.2 (7.5-14.7)
Karyotype (N/A/ND)	8/4/5	27/9/7
**Mutations (mutated/analyzed)**		
SRSF2	4/17 (23.5%)	21/43 (48.8%)
ASXL1	9/17 (52.9%)	27/43 (62.8%)
RUNX1	2/17 (11.7%)	9/43 (20.9%)
NRAS	5/17 (29.4%)	6/43 (13.9%)
KRAS	2/17 (11.7%)	7/43 (16.3%)
CBL	0/17 (0%)	3/43 (6.9%)

Supplementary Table 2: Independent cohort of CMML patients used for RT-qPCR analysis of *MIF* gene expression in sorted peripheral blood monocytes.

CMML	**TET2 wild type**	**TET2 mutated**
Number of patients	56	90
Mean age in years (range)	68.9 (30-87)	73.3 (55-90)
Sex ratio M/F	26/20	61/29
CMML 0/1/2	20/18/9	34/39/11
Proliferative/Dysplastic	23/30	39/49
Leucocytes, mean.10^9/L (range)	21.5 (3.6-119)	20.6 (3.0-141.9)
Monocytes, mean.10^9/L (range)	4.8 (1.0-28.8)	5.5 (1.0-59.7)
Platelet count, mean.10^9/L (range)	185.5 (18-560)	124.1 (13-553)
Hemoglobin level, mean g/dL (range)	11.3 (6.3-17.2)	12.1 (6.0-16.4)
Karyotype (N/A/ND)	25/12/19	54/14/22
**Mutations (mutated/analyzed)**		
SRSF2	11/55 (20.0%)	42/88 (47.7%)
ASXL1	16/56 (28.6%)	31/90 (34.4%)
RUNX1	7/56 (12.5%)	15/90 (16.7%)
NRAS	9/56 (16.1%)	14/90 (15.5%)
KRAS	7/56 (12.5%)	15/90 (16.7%)
CBL	5/56 (8.9%)	12/90 (13.3%)

Supplementary Table 3: Characteristic of CMML patients whose bone marrow aspirates were used to quantify MIF.

CMML	TET2 wild type	TET2 mutated
Number of patients	12	21
Mean age in years (range)	73.9 (58-84)	75.1 (59-86)
Sex ratio M/F	7/3/2	12/5/4
CMML 0/1/2	4/5/0	6/2/9
Proliferative/Dysplastic	6/2	12/6
Leucocytes, mean.10^9/L (range)	23.7 (9.4-50.3)	26.1 (5.7-110.4)
Monocytes, mean.10^9/L (range)	6.8 (1.5-27.6)	8.4 (1-67)
Platelet count, mean.10^9/L (range)	176.1 (44-461)	120.7 (27-394)
Hemoglobin level, mean g/dL (range)	10.7 (7.7-15.5)	11.0 (7.2-15.2)
Karyotype (N/A/ND)	6/0/6	13/4/4
**Mutations (mutated/analyzed)**		
SRSF2	3/12 (25%)	9/21 (42.8%)
ASXL1	8/12 (66.6%)	13/21 (61.9%)
RUNX1	2/12 (16.6%)	4/21 (19.0%)
NRAS	3/12 (25%)	3/21 (14.3%)
KRAS	0/12 (0%)	4/21 (19.0%)
CBL	3/12 (25%)	4/21 (19.0%)

Supplementary Table 4: Cohort of CMML patients whom sorted CD34 cells were analyzed by agilent arrays for gene expression**.**

**CMML**	**TET2 wild type**	**TET2 mutated**
Number of patients	13	9
Mean age in years (range)	75.5 (53-87)	71.3 (60-91)
Sex ratio M/F	10/3	7/2
CMML 0/1/ 2	5/6/2	4/4/1
Proliferative/Dysplastic	6/7	5/4
Leucocytes, mean.10^9/L (range)	41.4 (4.3-366.8)	25.4 (4.0-92.7)
Monocytes, mean.10^9/L (range)	9.1 (1.1-84.3)	10.3 (1.3-46)
Platelet count, mean.10^9/L (range)	241.4 (32-1050)	156.2 (18-335)
Hemoglobin level, mean g/dL (range)	11.0 (6.8-15.5)	12.2 (7.1-15.4)
Karyotype (normal/abnormal/not done)	7/6/0	8/1/0
**Mutations (mutated/analyzed)**		
SRSF2	7/13 (53.8%)	6/9 (66.7%)
ASXL1	8/13 (61.5%)	2/9 (22.2%)
RUNX1	1/13 (7.7%)	0/9 (0%)
NRAS	2/13 (15.4%)	3/9 (33.3%)
KRAS	1/13 (7.7%)	1/9 (11.1%)
CBL	2/13 (15.4%)	1/9 (11.1%)

Supplementary Table 5: Primers and antibodies used in this study.

RT-qPCR Primers	Sequences (5' - 3')
**RPL32**	
Forward	TGTCCTGAATGTGGTCACCTGA
Reverse	CTGCAGTCTCCTTGCACACCT
**PPIA**	
Forward	GTCGACGGCGAGCCC
Reverse	TCTTTGGGACCTTGTCTGCAA
**HPRT**	
Forward	GGACAGGACTGAACGTCTTGC
Reverse	CTTGAGCACACAGAGGGCTACA
**MIF**	
Forward	GAACCGCTCCTACAGCAAGCT
Reverse	TTGGCCGCGTTCATGTCG
**EGR1**	
Forward	TGATGTCCCCGCTGCAG
Reverse	GTCCATGGTGGGCGAGTG
**TET2**	
Forward	CCTGATACCATCACCTCCCATT
Reverse	CAGGCAGTGGGCTTCCAT
**ChIP qPCR Primers**	**Sequences (5' - 3')**
**MIF prom4 (R1)**	
Forward	AAGGGTGGCTCGACAGTCAA
Reverse	GCCAGAAGCCCCTCTCAAG
**MIF prom1 (R2)**	
Forward	CACCTGCCGCGATGACTAC
Reverse	CGCATGCGTGAGCTTGTG
**MIF prom2 (R3)**	
Forward	GCCTCCTGGCGACTAACATC
Reverse	CCACTTGGCGGCTAGAAATC
**MIF prom3 (R4)**	
Forward	GCGGGTCTCCTGGTCCTT
Reverse	GCACGTTGGTGTTTACGATGA
**Oligonucleotides Luciferase Assay**	**Sequences (5' - 3')**
**pGL3**	
Forward	CTTTATGTTTTTGGCGTCTTCCA
Reverse	CTAGCAAAATAGGCTGTCCC
**NFκB mut**	
Forward	GCAGGCGGTGATGGCCCCACTCGGG
Reverse	CCCGAGTGGGGCCATCACCGCCTGC

**EGR1 mut**	
Forward	AGCCGCAGCCTAATGGCGGCGGGGC
Reverse	GCCCCGCCCCCATTAGGCTGCGGCT
**$Sp1^p$ mut**	
Forward	TCCCCACTCGGTAGTGAGCCGCAGCC
Reverse	GGCTGCGGCTCACTACCGAGTGGGGA
**$Sp1^d$ mut**	
Forward	TCGCGGGGGCGACTCCTGGCGCCGG
Reverse	CCGGCGCCAGGAGCGCCCGCCA
**Antibodies ChIP experiments**	**Reference**
TET2 Ab1	C2 a gift of Dr Olivier Bernard
TET2 Ab2	sc-136926, Santa Cruz Biotechnology
P-S5 polymerase II	#39749, Active Motif
H3K4me3	#39159, Active Motif
H3K27me3	#39155, Active Motif
EGR1	sc-110, Santa Cruz Biotechnology
HDAC1	#39531, Active Motif
HDAC2	#39533, Active Motif
**Antibodies FACS experiments**	**Reference**
CD14-PE-Cy7	clone MϕP9, BD Biosciences (San Diego, CA)
CD15-PE-CF594	clone HI98, BD Biosciences (San Diego, CA)
CD16-APC-H7	clone 3G8, BD Biosciences (San Diego, CA)
CD34-FITC	cat 555821, BD Biosciences (San Diego, CA)
