# Supplementary material for: Macrophage migration inhibitory factor is overproduced through EGR1 in TET2low resting monocytes
Source: Commun Biol. 2022 Feb 3;5:110. doi: 10.1038/s42003-022-03057-w (PMC8814058; doi:10.1038/s42003-022-03057-w)
Supplement: Supplementary file 3 — Description of Additional Supplementary Files [file 42003_2022_3057_MOESM3_ESM.pdf]

Description of Additional Supplementary Files

File name: Supplementary Data 1
Description: RNAseq normalized counts

File name: Supplementary Data 2
Description: Control 1_EGR1_versus_Control 2_EGR1_common_peaks

File name: Supplementary Data 3
Description: CMML 1818_EGR1_versus_CMML 1900_EGR1_common_peaks

File name: Supplementary Data 4
Description: Controls_EGR1_versus_CMMLs_EGR1_common_peaks

File name: Supplementary Data 5
Description: Controls_EGR1_versus_CMML 1268_EGR1_common_peaks

File name: Supplementary Data 6
Description: log2Int Summarized CD34 samples

File name: Supplementary Data 7
Description: Source data underlying graphs and charts
